# Basecalling-free resistance gene identification using a hybrid transformer in raw nanopore signals

**DOI:** 10.3389/fmicb.2026.1748934

**Published:** 2026-02-18

**Authors:** Roman Jakubicek, Jevhenij Vorochta, Marketa Jakubickova, Matej Bezdicek, Martina Lengerova, Helena Vitkova

**Affiliations:** 1 Department of Biomedical Engineering, Brno University of Technology, Brno, Czechia; 2 Division of Clinical Microbiology and Immunology, Department of Laboratory Medicine, University Hospital Brno, Brno, Czechia; 3 Division of Clinical Microbiology and Immunology, Department of Laboratory Medicine, Faculty of Medicine, Masaryk University, Brno, Czechia

**Keywords:** antimicrobial resistance, convolutional encoder, floating window approach, *Klebsiella pneumoniae*, real-time detection, self-attention model, squiggle

## Abstract

Nanopore sequencing enables real-time access to raw signal data, which brings new possibilities for rapid genomic diagnostics. However, current workflows still primarily rely on basecalling, a computationally intensive step that slows subsequent analysis and limits real-time use. In addition, most current approaches that work with raw signals focus on simple read-level classification tasks and are not designed to detect and localize specific genes, particularly complex genomic features such as antibiotic resistance genes (ARGs). Here, we show that the hybrid convolutional-transformer model, NanoResFormer, can detect clinically relevant ARGs directly from raw nanopore signals without basecalling. The model captures both local and long-range signal patterns and employs a floating-window strategy to process inputs of varying lengths efficiently. In proof-of-concept experiments, NanoResFormer achieved a sensitivity of 92.6% and a precision of over 93%, with short latency, enabling real-time resistome profiling already during sequencing. The proposed approach, therefore, provides rapid access to crucial information, accelerating decision-making in clinical diagnostics and pathogen surveillance.

## 1 Introduction

Nanopore sequencing has emerged as a transformative technology for real-time, long-read analysis of DNA and RNA, eliminating the need for complex library preparation and enabling the continuous streaming of signal-level data as individual molecules pass through the nanopores (Wang et al., 2021). In a standard analytical workflow, raw current signals (referred to as “squiggles”) are first translated into nucleotide sequences through a process called basecalling. These sequences form the basis for downstream analyses, including alignment, variant calling, or gene annotation. However, basecalling is computationally demanding, often requiring GPU acceleration, which causes a delay in downstream analyses. Consequently, achieving genuine real-time data interpretation or diagnostics remains a significant challenge. Direct processing of raw nanopore signals makes it possible to bypass basecalling entirely and perform tasks such as squiggle classification or feature extraction already during sequencing. Although digital signal processing methods such as the Fourier transform or dynamic time warping might appear suitable for this purpose, their applicability is limited. In nanopore signals, individual current levels reflect k-mers rather than single nucleotides, and the signal is further distorted by noise and variable translocation speeds through the pore (Rang et al., 2018). As a result, traditional signal processing approaches are insufficient, and direct analysis of raw nanopore signals relies almost exclusively on machine learning methods.

In this context, recent advances in deep learning have enabled a paradigm shift in the field. One of the pioneering models, SquiggleNet, demonstrated the ability to classify nanopore reads directly from their electrical signals (e.g., distinguishing between reads from humans and bacteria) with high accuracy and minimal memory requirements, significantly accelerating decision-making compared to traditional alignment-based workflows (Bao et al., 2021). Building on this concept, the RawMap tool introduced CPU-efficient classifiers that utilize read prefixes in signal form, enabling real-time selective sequencing strategies such as read-until and the ejection of non-target reads (Sadasivan et al., 2023). Similarly, ReadCurrent employed a convolution-based architecture to achieve high-accuracy classification of target versus non-target DNA from raw nanopore signals in mixed datasets (human, yeast, bacteria, and viruses) while maintaining low computational requirements (Fan et al., 2024). Most recently, the Sigmoni tool extended this approach by enabling rapid multiclass classification of large pangenomes directly in the signal space, further demonstrating the potential of basecalling-free workflows for scalable genomic analysis (Shivakumar et al., 2024). Neural networks have also been applied to detect specific genomic regions directly in raw nanopore signals rather than classifying entire reads. NanoGeneNet, a hybrid convolutional-recurrent model, demonstrated accurate localization and classification of housekeeping genes used in multilocus sequencing typing (MLST) of *Klebsiella pneumoniae*, providing proof of concept for direct gene detection in signal space (Nykrynova et al., 2022).

A key application where direct signal-based analysis could have a significant impact is the rapid identification of antibiotic resistance genes (ARGs), as knowledge of antibiotic resistance profiles is essential for informed treatment decisions and effective infection control strategies. Unlike housekeeping loci, detecting ARGs is more complex. Resistance genes are highly diverse, may vary in copy number, and are often considerably longer. Thus, they must be recognized in their entirety to distinguish between closely related isoforms. Moreover, ARGs are not universally present in all genomes; thus, models must also reliably detect their absence. This variability in presence, copy number, and length means that the model must be able to recognize both small local patterns in the signal and broader dependencies that extend across thousands of signal samples.

For such complex tasks, transformer-based architectures (Vaswani et al., 2017) are well-suited. Initially developed for natural language processing, transformers offer distinct advantages when working with sequential data of varying lengths, including superior self-attention mechanisms and efficient parallelization. Compared to recurrent models like Long Short-Term Memory (LSTM) (Hochreiter and Schmidhuber, 1997), transformers excel at capturing long-range dependencies, an essential feature for identifying complete resistance genes that can span thousands of signal samples. In this context, the convolutional encoder serves to reduce dimensionality and extracts local motifs, while the transformer component models broader signal patterns corresponding to complete gene sequences.

In the present study, a hybrid transformer-based model was designed and tested to detect a panel of clinically relevant ARGs directly from raw nanopore signals. The selected genes cover several antibiotic classes, including β-lactamases (*blaSHV, blaOXA*), aminoglycoside-modifying enzymes (*aac, aph*), efflux pump genes associated with quinolone resistance (*OqxA, OqxB*), tetracycline resistance genes (*tetA, tetD*), and fosfomycin resistance genes (*fosA*) (Li et al., 2023). To evaluate this approach, raw nanopore sequencing data of *K. pneumoniae* were used, as these genes are frequently found in multidrug-resistant strains of this pathogen, which the WHO recognizes as a critical public health threat (Li et al., 2023). In proof-of-concept experiments, the transformer model achieved high sensitivity and specificity for gene detection in streaming mode, with minimal latency, making it suitable for real-time applications. By operating directly on raw signals and postponing or even bypassing basecalling, this approach enables the identification of resistance genes during the sequencing process itself. This approach paves the way for real-time resistome profiling in clinical workflows, offering clinicians earlier insights into resistance patterns, enabling more effective treatment selection, and reducing the risk of inappropriate empiric therapy and the spread of resistance in healthcare facilities. The proposed tool, called *NanoResFormer*, including all source code, is available on GitHub: BioSys-BUT/NanoResFormer.git.

## 2 Materials and methods

### 2.1 Sample collection and sequencing

A total of 61 *K. pneumoniae* isolates were collected between April 2018 and November 2023 from 13 departments at the University Hospital Brno (Brno, CZE). For DNA extraction, the DNeasy PowerSoil Pro Kit (Qiagen, Venlo, NL) was used, and DNA purity was measured using a NanoDrop spectrophotometer (Thermo Fisher Scientific, Waltham, MA, USA). The DNA concentration was checked using the Qubit 3.0 Fluorometer (Thermo Fisher Scientific, Wilmington, DE, USA). The sequencing library was prepared using the Rapid Barcoding Kit 96 V14 (Oxford Nanopore Technologies, Oxford, UK). Sequencing was performed using the PromethION 2 Solo sequencing platform (Oxford Nanopore Technologies, Oxford, UK) with R10.4.1 flow cells, and the obtained data were stored in POD5 format.

### 2.2 Signal dataset preparation

The signal dataset, comprising squiggles with and without target resistance genes, was prepared as follows. First, POD5 files were basecalled (using a super-accurate model) and demultiplexed with Dorado, integrated into MinKNOW (v23.11.5, nanoporetech.com/community). For each barcode, BAM files were converted to SAM format using Samtools (Danecek et al., 2021). Next, SAM files, together with POD5 files and FASTA files containing query genes, were processed using NANOSLAST (Jakubickova et al., 2025), which identifies squiggles containing target genes and their precise positions within the signal. Only squiggles containing the gene of interest that met empirically defined thresholds for minimum match length and alignment score, which reflect each gene's allelic variability, were stored. The thresholds range between 80%–90% of the maximum alignment score and 80–95% of the length of the searched gene (see Supplementary Table S1). Consequently, CSV files were generated containing nanopore signals with the genes of interest and the indices of their start and end points within the signals.

**Figure 1:**
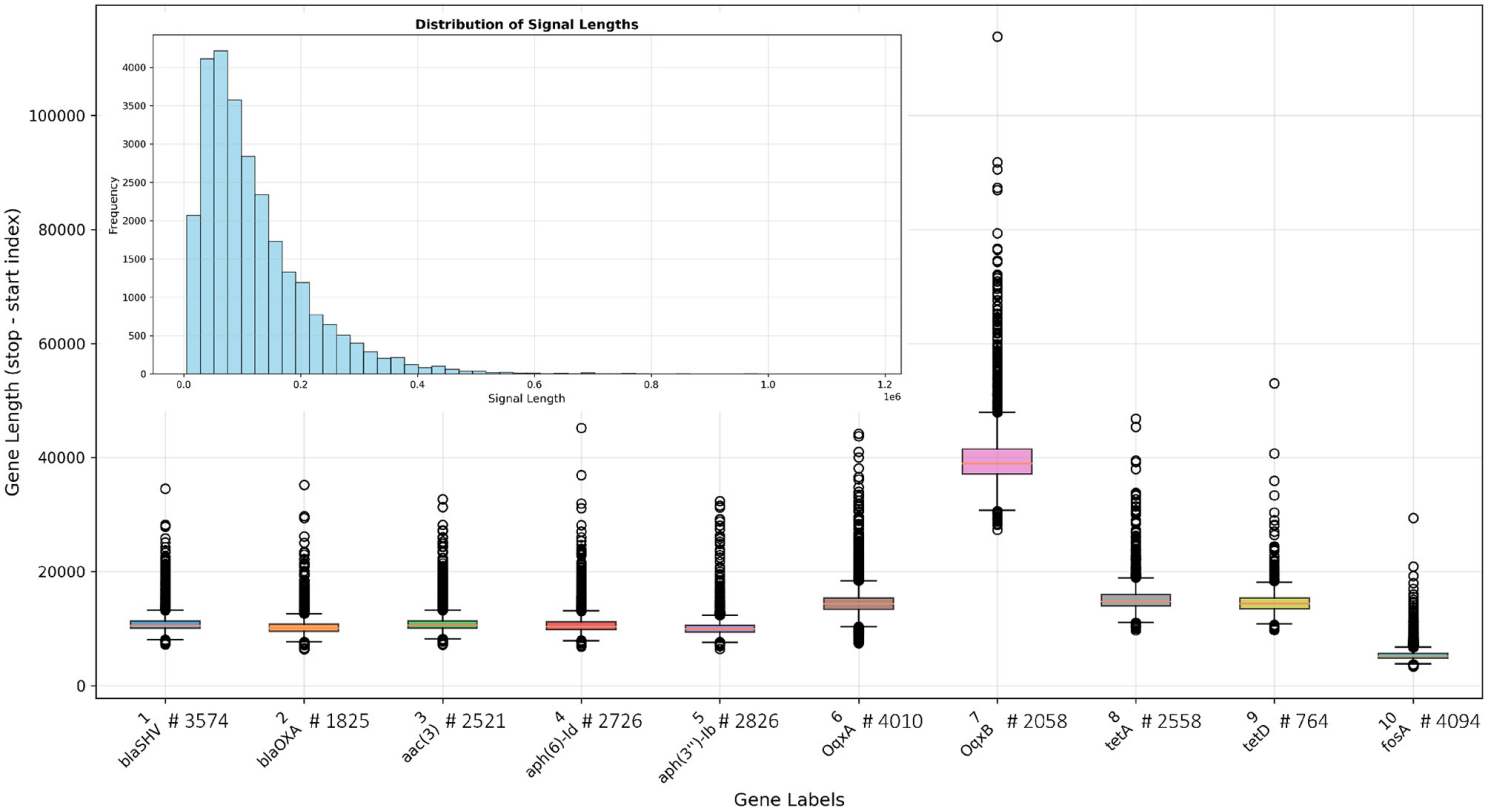
Box plots showing the distribution of individual gene lengths in the available signal database. Gene labels are displayed on the x-axis, with the corresponding number of genes indicated next to each label. The histogram in the upper-left corner illustrates the distribution of total reads (signals) across the database.

In total, 26,956 nanopore signals containing genes of interest were obtained (for detailed information, see Supplementary Table S2). The signal lengths ranged from 5,552 to 1.1 million samples, with average and median lengths of 120,590 and 95,361 samples, respectively. Nanopore signals exceeding 300,000 samples occurred only 1,397 times, accounting for 5.2% of the dataset. The mode signal length was 63,736 samples. The signal length histogram is shown in Figure 1. The length of the selected genes (in the signal form) was, on average, 13,341 samples, with a median of 10,812 samples. As shown in Figure 1, the *OqxB* gene has the longest signal segments, whereas the *fosA* gene has the shortest.

The obtained data were divided into training, validation, and test sets in a ratio of approximately 80:15:5, with the split performed at the isolate level (i.e., all signals from a given isolate were assigned to a single set). For batch learning, the training signals were randomly fragmented into segments using the data loader. Each segment was then assigned to one of the 11 classes (10 target genes plus a class with no gene of interest) based on its annotated gene content. Within each batch, segments containing gene information were randomly selected, ranging from 1 to the batch size (BS), and the batch was then supplemented with segments lacking any of the genes of interest. In addition, a database of signals without any genes of interest was created for the testing phase, corresponding to five percent of the total number of signals in the database.

### 2.3 Network architecture design

**Figure 2:**
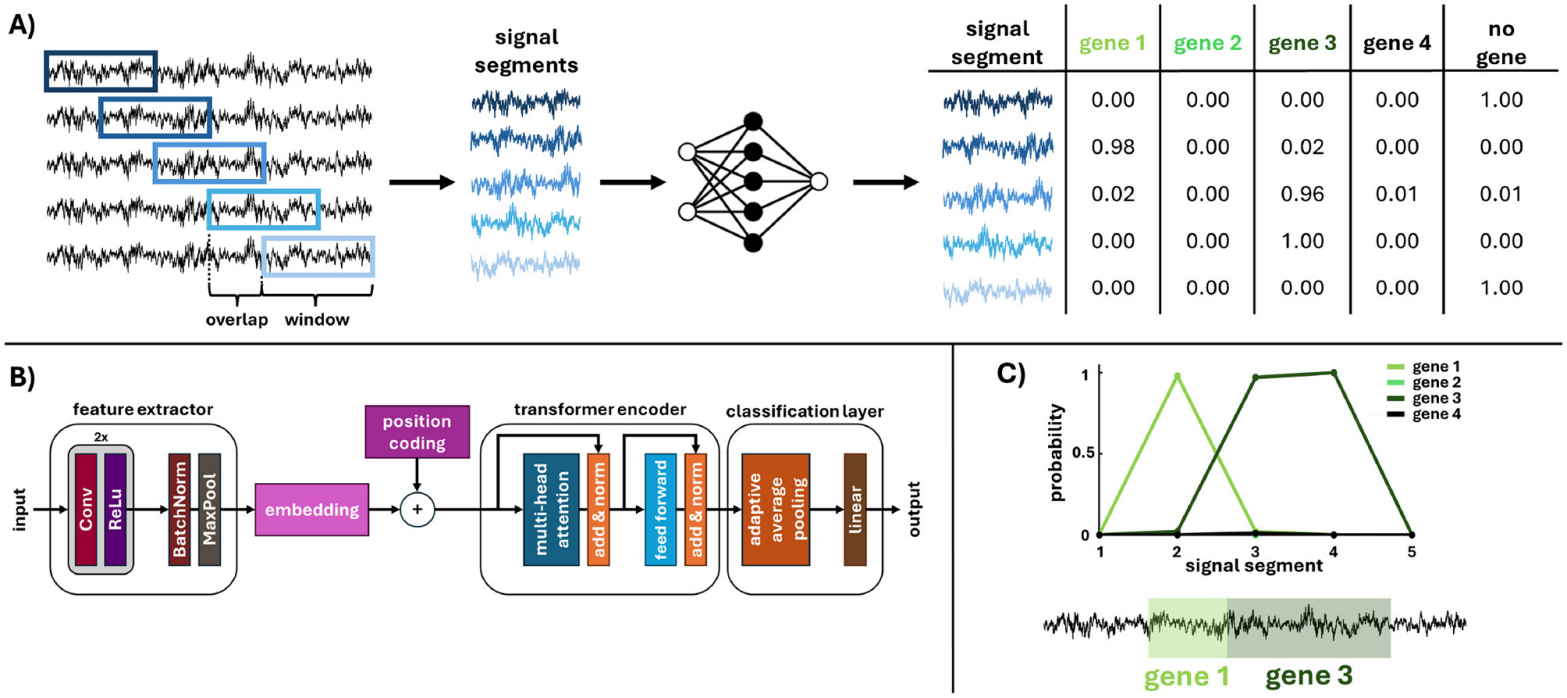
Graphical scheme of the proposed pipeline for gene detection in raw nanopore signals. **(A)** Overview of the floating-window approach: reads are divided into segments, processed by a neural network, and output as a classification table with segment-level probabilities. **(B)** Architecture of the proposed hybrid neural network, combining a CNN-based feature extractor with a classification transformer. **(C)** Example of network output: a series of class (gene) assignment probabilities for each segment, followed by backward rearrangement and interpolation of probabilities for each signal point. The real output is shown in Figure 6.

Given the nature of the processed signals described in Section 2.2, a hybrid network architecture was proposed, combining a convolutional neural network (CNN) subsampling followed by a transformer encoder block (see Figure 2B). The CNN encoder extracts relevant features and substantially downsamples the signals (see Table 1). The resulting feature representation is then processed by the transformer encoder, which analyses the signal and assigns it to one of 11 classes via a fully connected classifier.

**Table 1:** Summary table for three models, including the characteristics of the proposed models (number of features and level of sub-sampling), the results achieved on the testing database using the floating-window method, and the time complexity expressed in hours per million signals.

**Model**	**# feat**.	**Sub-samp**.	**Sensitivity (%) ±CI95**	**Precision (%) ±CI95**	*FPR (%) ±CI95	**Time (h) ±CI95**
Low	8	64 ×	72.8 ± 2.5	60.4 ± 2.0	30.5 ± 2.4	**5.74** **±0.16**
Middle	64	64 ×	92.6 ± 1.3	**93.1** **±1.4**	**3.8** **±1.0**	6.28 ± 0.40
High	128	16 ×	**94.6** **±1.2**	91.1 ± 1.6	16.0 ± 2.0	11.08 ± 1.36

Sensitivity and accuracy values were obtained as averages for ten genes. The false positive rate (FPR) values were calculated only from signals without the gene of interest. Confidence interval (CI95) values are expressed as ± margin of error with level 95% of confidence from 1,000-fold bootstrapping.

^*^False positive rate for dataset of signals with no gene of interest.

The best achieved values among the three models are highlighted in bold.

The CNN-based extractor comprises a series of extraction blocks, each comprising two convolutional layers with a 3 × 3 kernel size, followed by a ReLU activation function. The number of blocks varies for different network variants (see Table 1). The ablation study of the number of features and layers in the encoder part was simplified; thus, three model variants were designed and evaluated: Low, Middle, and High (see Table 1). These variants differ in the degree of signal downsampling and the number of extracted features. The CNN encoder produces a sub-sampled signal whose reduced length corresponds to the maximum number of tokens in the transformer block, with each token length determined by the number of extracted features.

An additional ablation study was conducted for the transformer part of the model, focusing on the number of heads and layers, which mainly affect the trade-off between sensitivity and precision. These parameters also had a significant impact on FPR values. The results of this ablation study are shown in Supplementary Figure S1. Based on the achieved evaluation metrics, a configuration with two layers and one head was chosen for the final model.

### 2.4 Training details

The network was implemented in Python 3.12 with PyTorch (v2.8). Given the multiclass nature of the classification task, the cross-entropy loss function was chosen, along with the ADAM optimization algorithm, using β_1_ = 0.900 and β_2_ = 0.999. Based on preliminary experiments, the number of epochs was set to 250, and the best model was saved according to validation performance to prevent both overfitting and underfitting.

An ablation study to determine the optimal learning hyperparameters for each model was conducted using a sophisticated Bayesian optimization method, specifically, the learning rate (LR), batch size (BS), and dropout probability (DP). The resulting parameters were consistent across all models: LR = 0.001, BS = 64, and DP = 0.0. This optimization was performed using the Bayesian Optimization package (Nogueira, 2014) with 20 iterations and 5 initialization points. A 5-fold cross-validation yielded an average classification accuracy of 81.74% (SD 2.53%), indicating low sensitivity to random sampling and suggesting robustness to variations in data balance and variability.

Basic data processing and testing were performed on a workstation equipped with an Intel Core i9 3.70 GHz processor, 64 GB of RAM, a 2 TB M.2 SSD, and an EVGA GeForce RTX 3090 graphics card with 24 GB of GDDR6 memory. For more computationally intensive tasks, such as network training and Bayesian hyperparameter optimization, resources provided by the e-INFRA CZ project (ID:90254) were used.

### 2.5 Training classification performance

**Figure 3:**
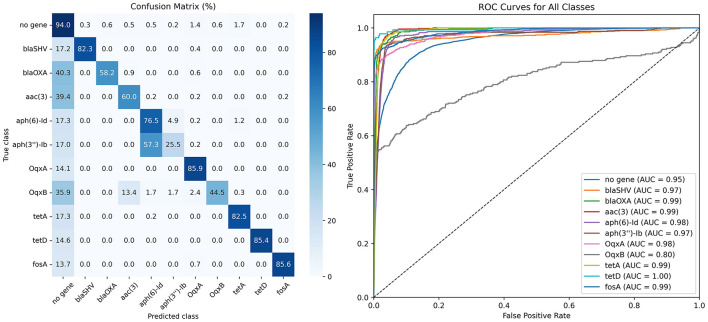
Results showing the success rate of the classification of reads by the trained Middle model on the validation set as a purely classification task (without floating window access). The confusion matrix for individual classes (genes) is shown on the **left**, and the ROC analysis is shown on the **right**.

The network was trained as a multi-class classifier, assigning signal segments to one of 11 predefined classes. For this classification task, a confusion matrix was computed on the validation set, and the ROC analysis for the Middle model is shown in Figure 3 (see Supplementary Figures S2, S3 for the remaining models). During training, the model achieved an average validation accuracy of 87.5%.

The ROC analysis indicates that the *OqxB* gene (gray curve in Figure 3) is particularly challenging to classify (AUC = 0.80), likely due to its close genomic proximity to *OqxA*. Another contributing factor may be the length of the *OqxB* gene segments, which are the longest among all genes, as shown in Figure 1. Furthermore, a substantial portion of these segments exceeds our window length of 40,000 samples, indicating that partial gene fragments occur more frequently in this gene than in others, which may be the primary reason for the lower accuracy.

Nevertheless, the overall performance improves markedly when using a floating window with a defined overlap, which enables prediction boosting for local classification and supports multiple localization tasks.

### 2.6 Floating window approach for continuous classification

**Figure 4:**
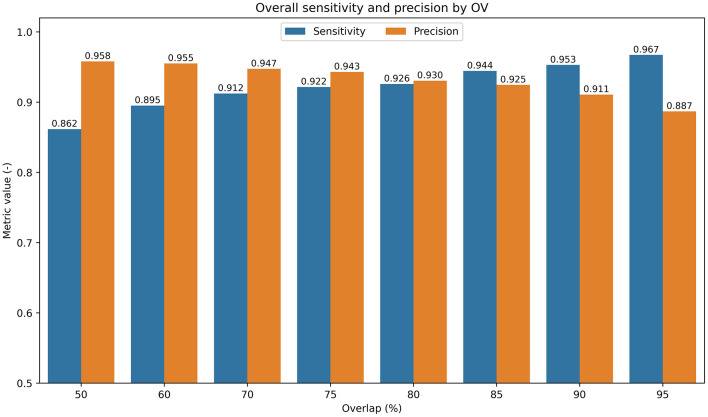
Bar chart showing the average sensitivity (blue) and precision (orange) for all genes, including genes that are not of interest, in the Middle model predictions on the test dataset at different floating window overlap settings.

To address the variability and extreme length of signals, an approach utilizing a floating window (shown in Figure 2A) with a window size of 40,000 samples was proposed. The window size covers most gene lengths and allows for a defined extent. Each segment within the floating window is classified; for every shift, the network outputs class-assignment probabilities for the segment. Before being processed by the network, the signal is extended (padded) at both ends by half the window length to mitigate edge effects. The chosen overlap determines the density of classified positions in the signal, which are then interpolated across all signal samples (an example of such a function is shown in Figure 2C). This approach enables the approximation of the gene location, with detection resolution influenced by both the overlap and the window size. However, precise gene positioning is beyond the scope of this study, which focuses solely on gene identification within reads. This process yields 11 probability values per signal sample, corresponding to the predefined classes. The final decision on gene presence or absence is based on selecting the class with the highest probability value for each signal sample. As part of the ablation study performed during the development of the sliding window approach, the impact of the window overlap percentage on detection performance was evaluated, as shown in Figure 4.

## 3 Results and discussion

Three different models with varying capacities and subsampling rates were designed to evaluate gene detection performance in a continuous detection regime (see Table 1 for their parameters and time complexity). Models were evaluated on a test dataset comprising 5% of the available data (i.e., 1,343 signals). Sensitivity and precision metrics, summarized in Table 1, were calculated to reflect detection performance. The test dataset was also expanded to include signals that did not contain any of the genes of interest in the same quantities as the gene signals. The false positive rate (in Table 1) was calculated, reflecting the number of false detections of genes that are not present. The Middle model, achieving almost 93% sensitivity, was selected for further testing as a compromise between performance and computational complexity.

**Figure 5:**
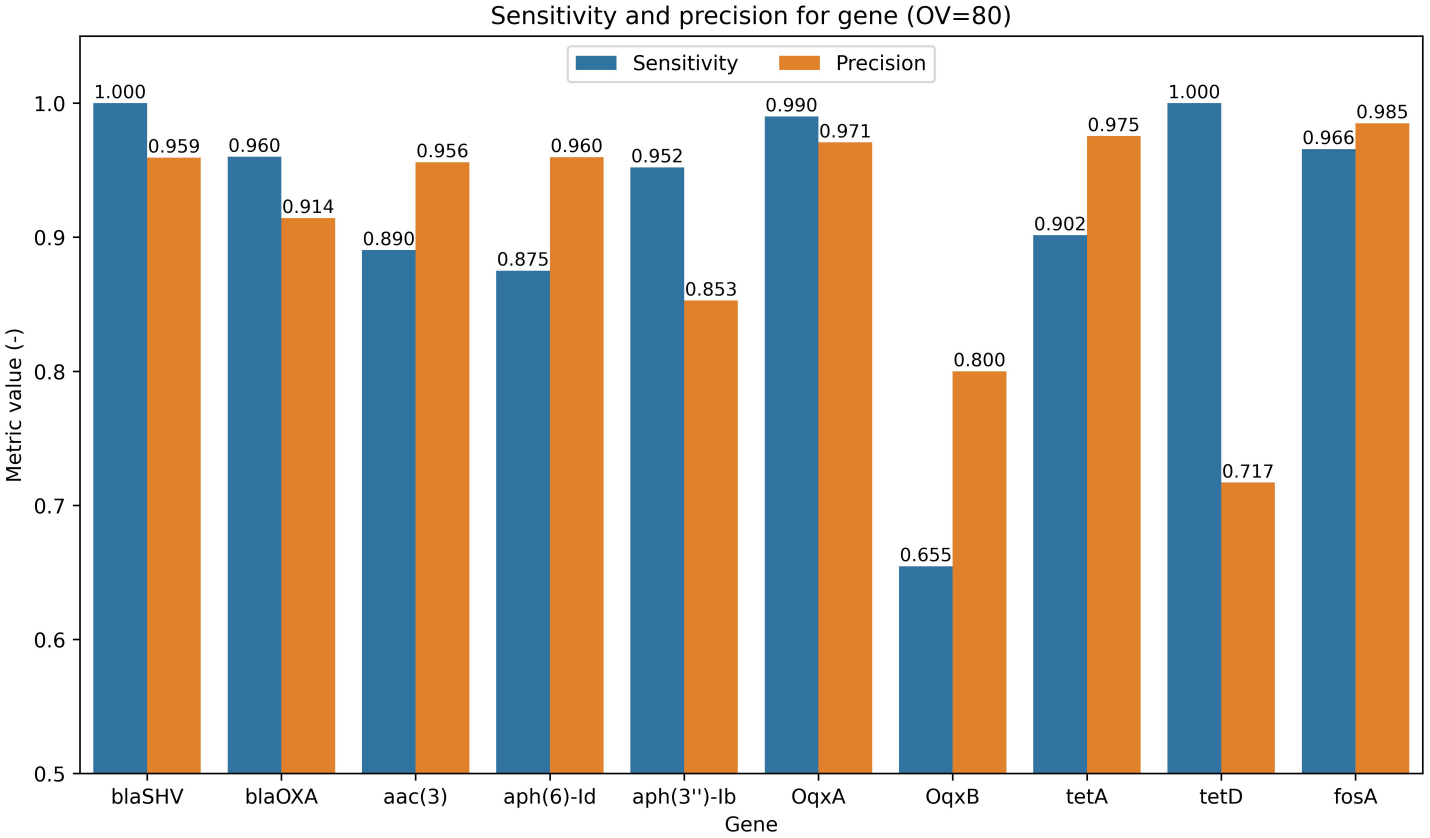
Bar chart showing the sensitivity (blue) and precision (orange) for individual gene identification in reads using the floating-window approach with the Middle model on the test dataset.

Detailed results for each gene (Figure 5) show a high sensitivity value (92.6% on average), confirming the model's ability to correctly identify genes of interest when present. Furthermore, the achieved precision value indicates that 93.1% of all detections were correctly identified. When the gene was not present in the signal, the model predicted only 3.8% false-positive detections of the gene's presence. The lowest value of metrics was observed for *OqxB* and *tetD*, at 65.5% and 71.7%, respectively. The lower sensitivity value for the *OqxB* gene may be due to its greater length compared to that of other genes. Approximately half of the instances of this gene exceed the size of our window, meaning the model often sees only part of it, potentially leading to inconsistent detection. The main cause of the low precision value for the *tetD* gene may be low data variability due to the limited number of instances of this gene in our dataset (764 occurrences of the gene; see Supplementary Table S2).

### 3.1 Analysis of success rate detection

Introducing a floating window with overlapping acts as a form of boosting, enabling multiple classifications of overlapping signal regions. This approach eliminates false detections caused by the possible inclusion of an incomplete gene region in the currently classified segment. The effect of boosting amplifies with increasing window overlap; however, it also increases computational complexity, as the signal is divided into multiple segments. According to the graph in Figure 4, there is an apparent natural increase in sensitivity; however, the probability of false detection of selected genes in similar regions is also increased, thereby reducing precision. The compromise with the highest average success rate seems to be an 80% overlap.

The possible inclusion of only part of a gene in a floating window is a natural effect that can affect the success of identification. The graph in Supplementary Figure S4 shows the dependence of the average identification sensitivity (calculated across all ten genes) on the proportion of the gene covered by the floating window, revealing an exponential trend. As the model may rely on different informative regions for different genes and cases, it is therefore unsurprising that the sensitivity reaches its maximum only when the entire gene is contained within the floating window. When only half of the gene is present in the window, the sensitivity decreases to 18%. Problems may thus arise when a gene lies at the end of a nanopore signal and is therefore not fully captured. However, since each strain is sequenced at an average depth of ~100 × , other reads typically contain the full gene, effectively mitigating this limitation.

### 3.2 Multiple gene occurrences

**Figure 6:**
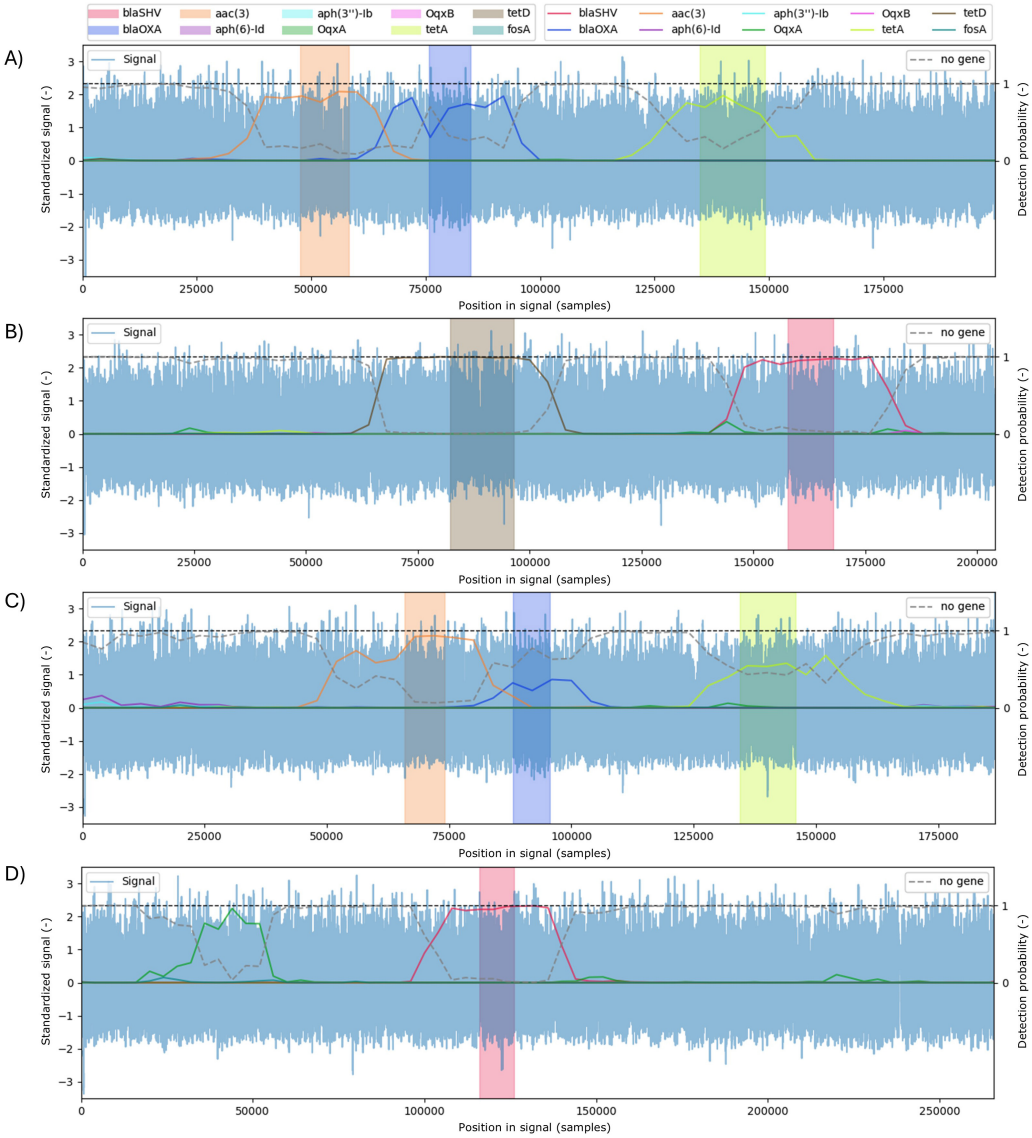
Examples of four reads (signals in blue, standardised using *z*-score normalisation) illustrating multiple gene detections. The graph displays linearly interpolated probability curves for all 10 gene classes plus the “no gene” class (grey dotted line). Colour-coded columns indicate ground-truth gene regions identified by BLAST. **(A)** Correct identification of three genes; **(B)** correct identification of two genes; **(C)** false negative for the *blaOXA* gene (blue), where the probability assigned to the “no gene” class is higher; **(D)** false positive case, where the model also detects the *OqxA* gene (green).

ARGs may occur multiple times within a bacterial genome, and similar genes often cluster in close proximity, complicating detection. Therefore, classifying entire read alone is insufficient; the floating window approach offers a new perspective on gene detection, even for multiple occurrences within a single read (signal). For each floating window position, probabilities for all gene classes, including the no gene category, are computed, and the class with the highest probability is selected as a final decision. Figure 6 illustrates examples of four signals with multiple occurrences of ARGs. In Figure 6C, a false negative case for the *blaOXA* gene (blue region) is observed, where the predicted absence probability exceeded the presence probability, despite BLAST confirming the gene's presence. This is despite the fact that, according to BLAST detection, the gene is present. Conversely, in the next signal (Figure 6D), *OqxA* shows a high predicted probability despite local alignment failing to detect the gene, with nucleotide sequence identity at only approximately 50%. No such false detections occur in the High model, suggesting that the false detections may be caused by information loss at higher subsampling rates introduced by the encoder or by lower network capacity, making it more difficult to identify slight changes in the signal pattern.

### 3.3 Computational time analysis

**Figure 7:**
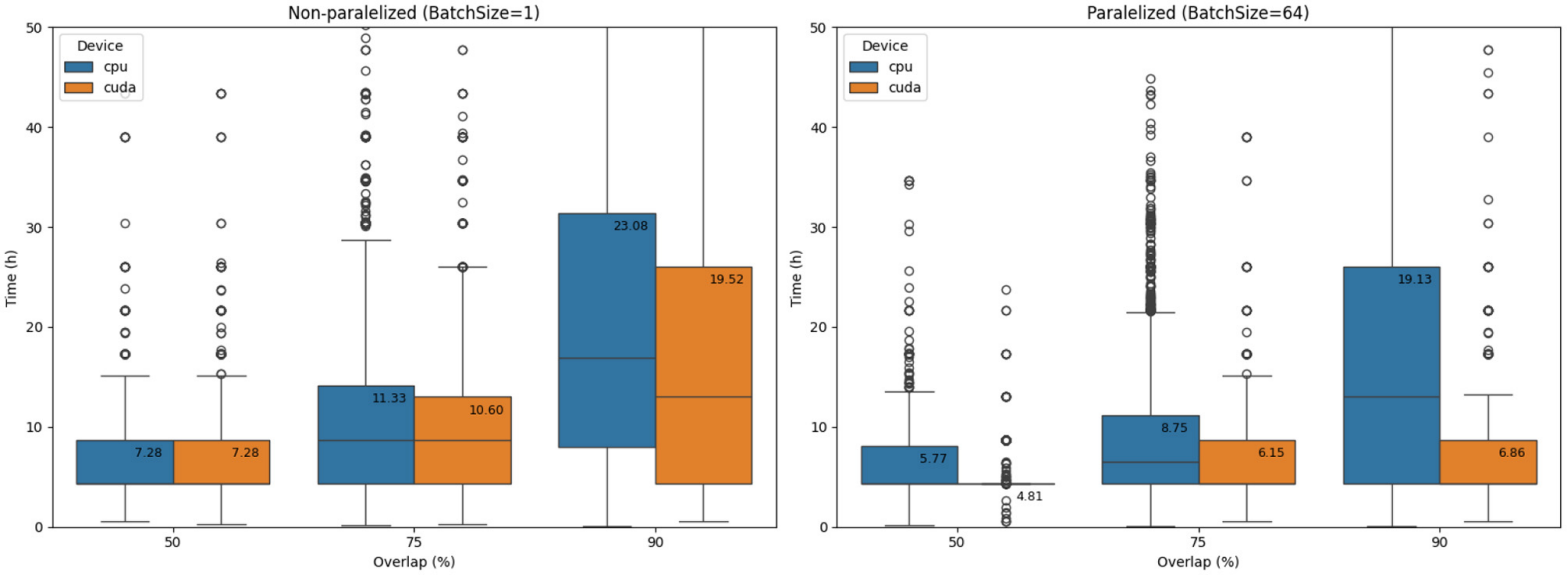
Comparison of the time complexity of the Middle model depending on the floating-window overlap and the use of parallelization on CPU and GPU devices. The graph on the **left** shows results without batch parallelization, and the graph on the **right** shows results with batch parallelization. Times are converted to hours per million signals (test dataset) and represent the average of ten measurement repetitions.

The time complexity of the selected model was evaluated on a test dataset under CPU and GPU configurations, with and without parallelization. Figure 7 illustrates how time complexity varies with the overlap parameter in the floating window approach. GPU batch processing offers substantial parallelization benefits, reducing computation time to approximately 6 h per million reads. Scaling to a larger number of classes (up to 1,000 gene types) is expected to minimally impact computational complexity, thanks to the network's architecture. Thus, the proposed model shows strong potential for detecting additional resistance-related genes, provided that the annotated dataset is expanded and the model is retrained.

### 3.4 Basecalling-free approach in practical application

**Figure 8:**
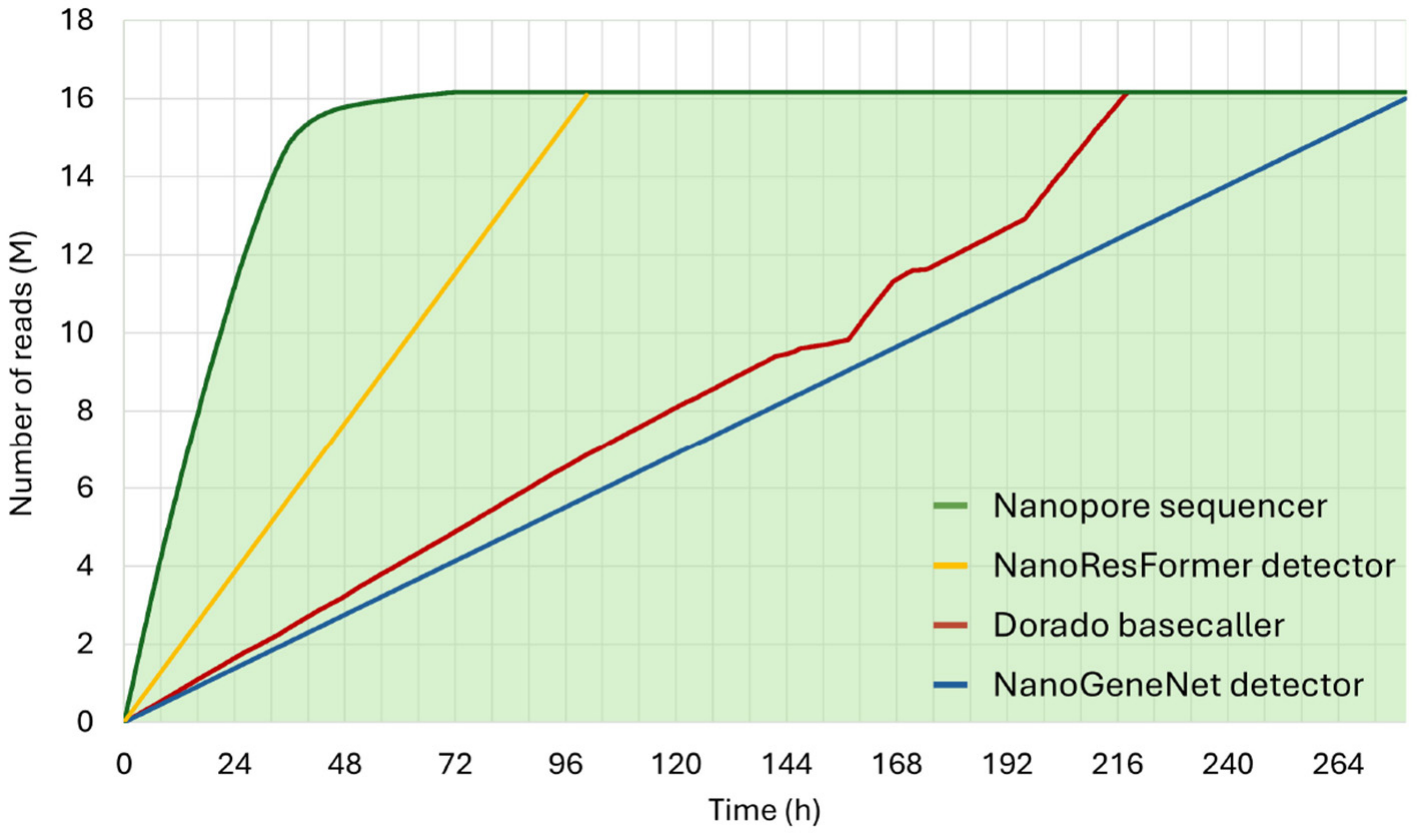
Cumulative read counts from a run on the PromethION 2 Solo device (61 *K. pneumoniae* genomes). The green curve shows the number of reads generated during sequencing; the red curve shows the basecalling process (Dorado integrated in MinKNOW, super-accurate model); the yellow curve shows the number of reads processed by NanoResFormer, and the blue curve shows the number of reads processed by NanoGeneNet, both for gene detection and determined based on computational time analysis.

Nanopore read processing traditionally relies on basecalling, where raw signals are converted into nucleotide sequences before downstream analysis. This conversion introduces substantial delays in the analytic workflow, as shown in Figure 8, which depicts cumulative read processing over time. Figure 8 reflects data from the sequencing run described in Section 2.1. Sequencing and basecalling statistics were extracted from MinKNOW logs, while NanoResFormer performance metrics were derived from computational time analysis (Section 3.3).

As shown in Figure 8, after 48 h of sequencing, only 20% of reads have been basecalled. Moreover, on the basecalled data, other tools capable of ARG identification need to be applied, further extending the time required to obtain crucial results. In contrast, our basecalling-free approach enables real-time direct analysis of raw signals, including AMR gene detection during sequencing. Within the first six h of sequencing, over one million reads can be screened with >92% sensitivity. The full sequencing run can be processed in approximately 96 h, less than half the time required for basecalling (216 h).

While basecalling remains necessary for some downstream applications, rapid ARGs identification via a basecalling-free strategy offers clear clinical benefits. Using Bernoulli distribution-based calculations and considering the model's sensitivity, the minimum number *n* of reads required to ensure that at least one of the resistant genes is included in the random selection with the specified level of confidence has been estimated. Using the lowest observed gene frequency (*p*_*min*_ = 0.0298%, Supplementary Table S2) and the formula *n* = *ln*(1 − 0.999)/*ln*(1−*p*_*min*_), it has been calculated that 24,586 reads are required to achieve 99.9% confidence in detecting at least one target gene. Given NanoResFormer's processing speed (Section 3.3), the first resistant gene is likely to be detected within 9.3 min. If no gene is found during this time, the absence can be inferred. If detected, processing an additional 805,545 reads (~5.2 hours) ensures 99.9% confidence that all resistance genes present are identified.

### 3.5 Comparison with NanoGeneNet

Due to the lack of similar published algorithms for the same task, our proposed approach is primarily compared with NanoGeneNet (Nykrynova et al., 2022), which, as mentioned in the Introduction, uses a combination of LSTM and CNN. This model was originally evaluated for the classification of seven MLST genes in nanopore signals, achieving a sensitivity of 92.0%. Although our model was designed to detect a different set of genes (10 resistance genes), it achieved very similar results for the same task (i.e., gene identification) on comparable nanopore signals generated using a newer sequencing chemistry (R10 instead of R9), with a sensitivity of 92.6%.

When comparing inference times, transformer-based models demonstrate a clear advantage thanks to their parallelization capabilities and efficient batch processing. The experiment shown in Figure 8, where both tools were evaluated under identical conditions, i.e., on the same hardware and nanopore signal testing database, confirmed this advantage, as NanoGeneNet was up to three times slower at identifying nanopore signals. This performance gap highlights transformer models' potential and their suitability for the real-time signal processing tasks addressed in this work.

As noted by the authors of NanoGeneNet (Nykrynova et al., 2022), training LSTM-based models is challenging and not entirely trivial, often requiring sequential training to mitigate the vanishing and exploding gradient problem. In contrast, transformers enable single-shot training, which makes it feasible to release, in future work, a user-friendly tool that enables users to retrain the model on new or custom nanopore signals.

The final area of comparison is the ability to detect multiple gene occurrences within a single signal. NanoGeneNet does not support this functionality because it uses global signal classification, albeit with localization. In our proposed tool, the implementation of a floating-window approach enables local classification, thereby detecting multiple gene occurrences within a single read.

### 3.6 Limitations

Although this study demonstrates the feasibility of transformer-based resistance gene detection from squiggles, several limitations must be addressed before clinical implementation. The current model was trained on a limited set of resistance genes, restricting its clinical applicability. Expanding the model to cover the comprehensive resistance gene panel required for clinical diagnostics poses significant challenges. Adding more gene classes directly to the existing architecture may be constrained by memory limitations and could reduce classification accuracy due to increased model complexity. A hierarchical modeling approach could be implemented to address this limitation through a two-tier classification system: broad-spectrum models would identify the presence of resistance gene families, followed by specialized models that resolve specific allelic variants when required. This strategy optimizes computational efficiency by avoiding simultaneous searches for all possible alleles and focusing only on those detected during the initial screening, thus making real-time implementation more feasible.

Another limitation may arise during the preparation of training data. Current labeling relies on BLAST-based alignment, which can introduce systematic bias. Specifically, highly variable alleles may evade detection yet remain recognizable to the transformer model, potentially causing false-positive classifications. Furthermore, these tools may not reliably identify newly emerged or significantly mutated variants of resistance genes that the model could still detect.

A further challenge lies in the diversity of sequencing data used to train the model. Training datasets should ideally span multiple library chemistries, flowcell types, and even different sequencing platforms to enhance robustness and generalization. However, creating such a diverse dataset is challenging in practice. Nevertheless, the dataset used in this study is based on the latest R10 chemistry, which is currently the most widely used and serves as the de facto standard in Oxford Nanopore Technologies (ONT) workflows.

To handle over-length reads that transformers cannot efficiently process at their original length, a floating-window approach has been proposed. This method enables the processing of variable-length signals, including extremely long ones, and improves detection success rates. Transformers complexity scales quadratically with token (window length) count; therefore, a 40,000-sample floating window was chosen, covering the lengths of all nine genes except *OqxB* (average length of 40,412 samples). Nevertheless, this gene was included in the study as it represents a borderline case in terms of the length of successful gene identification. Despite its borderline length, *OqxB* identification achieved 66% sensitivity. However, the transformer network cannot capture the entire signal context, and enlarging the window would significantly increase computational time. Overlapping floating windows partially mitigate this limitation, but it remains a practical concern.

## 4 Conclusion

ONT sequencers, owing to their portability and growing availability, are emerging as natural candidates for deployment as comprehensive diagnostic platforms rather than simple sequencing devices in clinical microbiology. To realize this vision, diagnostic evaluation must be integrated as an inherent part of the sequencing process. Because nanopore sequencing produces data continuously over tens of hours, real-time diagnostics should ideally commence immediately after each read is measured, long before the sequencing run is complete. However, conventional workflows rely on computationally intensive basecalling, resulting in substantial delays and requiring high-performance hardware.

Our results demonstrate that clinically relevant information can be extracted directly from raw current signals (squiggles), eliminating the need for basecalling. The proposed hybrid convolutional–transformer model, NanoResFormer, achieved 92.6% sensitivity with a 3.8% false-positive rate for resistance gene detection. Furthermore, it can screen one million reads in just 6.3 h, approximately half the time required for basecalling alone. Although basecalling remains essential for downstream applications such as genome assembly or variant analysis, our findings show that for targeted diagnostic tasks, a basecalling-free strategy is faster and sufficiently informative. Statistical modeling suggests that a preliminary diagnosis can be achieved within nine minutes of sequencing, as at least one resistant gene can be detected with 99.9% confidence after analyzing only 24,000 reads. Although detecting resistance genes does not, by itself, confirm phenotypic resistance, this work demonstrates the feasibility of signal-level diagnostics performed during sequencing.

Future work will focus on optimizing the approach for continuous real-time processing, including adaptive handling of incoming data streams and expanding the model to a broader spectrum of resistance genes. Further reductions in computational demands may be achieved through efficient transformer variants and optimized data management pipelines. As part of publishing easy-to-use software tools on GitHub, we would also like to create a simple module for user retraining on new or custom data. Ultimately, integrating models such as NanoResFormer directly into ONT sequencing devices could transform them into self-contained diagnostic systems, delivering actionable resistance information in real time and enabling rapid, on-site clinical decision-making.

## Data Availability

NanoResFormer is openly accessible on GitHub (BioSys-BUT/NanoResFormer.git). All POD5 sequencing data used in this study have been uploaded to the Czech National Repository and are available via the following DOI: 10.48700/datst.dj8ys-a4r49.
